# Microsatellite instability analysis in hereditary non-polyposis colon cancer using the Bethesda consensus panel of microsatellite markers in the absence of proband normal tissue

**DOI:** 10.1186/1471-2350-7-5

**Published:** 2006-01-20

**Authors:** Sergio G Chialina, Claudia Fornes, Carolina Landi, Carlos D de La Vega Elena, Maria V Nicolorich, Ricardo J Dourisboure, Angela Solano, Edita A Solis

**Affiliations:** 1 Histocompatibility and Molecular Biology Laboratory. Italian Hospital"Garibaldi". Rosario Santa Fe. Argentina; 2 Laboratory ACDM-Instituto Alexander Fleming, Buenos Aires, Argentina

## Abstract

**Background:**

Hereditary non-polyposis colon cancer (HNPCC) is an autosomal dominant syndrome predisposing to the early development of various cancers including those of colon, rectum, endometrium, ovarium, small bowel, stomach and urinary tract. HNPCC is caused by germline mutations in the DNA mismatch repair genes, mostly hMSH2 or hMLH1.

In this study, we report the analysis for genetic counseling of three first-degree relatives (the mother and two sisters) of a male who died of colorectal adenocarcinoma at the age of 23. The family fulfilled strict Amsterdam-I criteria (AC-I) with the presence of extracolonic tumors in the extended pedigree. We overcame the difficulty of having a proband post-mortem non-tumor tissue sample for MSI testing by studying the alleles carried by his progenitors.

**Methods:**

Tumor MSI testing is described as initial screening in both primary and metastasis tumor tissue blocks, using the reference panel of 5 microsatellite markers standardized by the National Cancer Institute (NCI) for the screening of HNPCC (BAT-25, BAT-26, D2S123, D5S346 and D17S250). Subsequent mutation analysis of the hMLH1 and hMSH2 genes was performed.

**Results:**

Three of five microsatellite markers (BAT-25, BAT-26 and D5S346) presented different alleles in the proband's tumor as compared to those inherited from his parents. The tumor was classified as high frequency microsatellite instability (MSI-H). We identified in the HNPCC family a novel germline missense (c.1864C>A) mutation in exon 12 of hMSH2 gene, leading to a proline 622 to threonine (p.Pro622Thr) amino acid substitution.

**Conclusion:**

This approach allowed us to establish the tumor MSI status using the NCI recommended panel in the absence of proband's non-tumor tissue and before sequencing the obligate carrier. According to the Human Gene Mutation Database (HGMD) and the International Society for Gastrointestinal Hereditary Tumors (InSiGHT) Database this is the first report of this mutation.

## Background

Hereditary non-polyposis colorectal cancer (HNPCC) is an inherited syndrome predisposing to the early development of cancers of colon, rectum, endometrium, ovarium, small bowel, stomach and urinary tract [1,2].

Since there are no premonitory signs of susceptibility to HNPCC, family history has been the primary method for identifying patients at risk. Defined by the International Collaborative group on HNPCC, the typical HNPCC family fulfill the following criteria (referred to as the Amsterdam-I criteria [3]): 1. Three or more relatives with histologically verified colorectal cancer, one of whom is a first-degree relative of the other two; 2. Colorectal cancer affecting at least 2 successive generations; and 3. At least one relative diagnosed with colorectal cancer under the age of 50. The fulfillment of these criteria prompted further genetics investigations. More recently it has been revised to take into account the prevalence of extracolonic cancer in certain HNPCC families [4].

This autosomal dominantly inherited disorder is caused by germline mutations in genes coding proteins responsible for the repair of DNA replication errors, which are referred to as DNA mismatch repair (MMR) genes [5]. DNA mismatch repair machinery plays a critical role in genomic stability, including correction of mispaired bases associated with DNA replication and recombination. Germline mutations in one allele of any of these genes followed by the somatic loss or inactivation of the wild-type allele leads to a defective mismatch repair mechanism. The current "gold standard" for assessing tumor DNA MMR activity is molecular microsatellite instability (MSI) testing. In most cases, it involves extracting DNA from both tumor and normal tissue. The DNA is subjected to polymerase chain reaction (PCR) amplification of five or more different chromosomal loci that compare "microsatellites", running the PCR products through a gel to separate DNA fragments by size, comparing the tumor-normal pairs, and scoring for differences between the two. Instability at two or more out of five markers defines a tumor as MSI-H and prompts further analysis, as sequencing of DNA MMR genes. A number of them have been associated with HNPCC, including *hMSH2*, *hMLH1, hPMS1, hPMS2, hMSH3*, and *hMSH6*. Most of the HNPCC families in which mutations have been identified involved *hMSH2 *and *hMLH1 *genes [6].

A much less labor-intensive alternative method used to prescreen high-risk individuals for further germline mutation analysis is immunohistochemistry (IHC) testing for MLH1 and MSH2 expression. IHC testing may identify which gene to target for analysis.

We describe MSI testing in the absence of proband non-tumor tissue using the Bethesda consensus panel (mononucleotide repeats BAT25 and BAT26, and dinucleotide repeats D2S123, D5S346, and D17S250) and we report a novel *hMSH2 *germline mutation found in the family.

## Methods

### Patients

Three first-degree relatives (mother and two sisters) of a male who died of poorly differentiated colorectal adenocarcinoma at age 23 contacted us for genetic counseling. A detailed family and medical history was obtained through interview with the proband relatives and their consent for release of medical records and use of the pathological tissue blocks still available.

**Figure 1:**
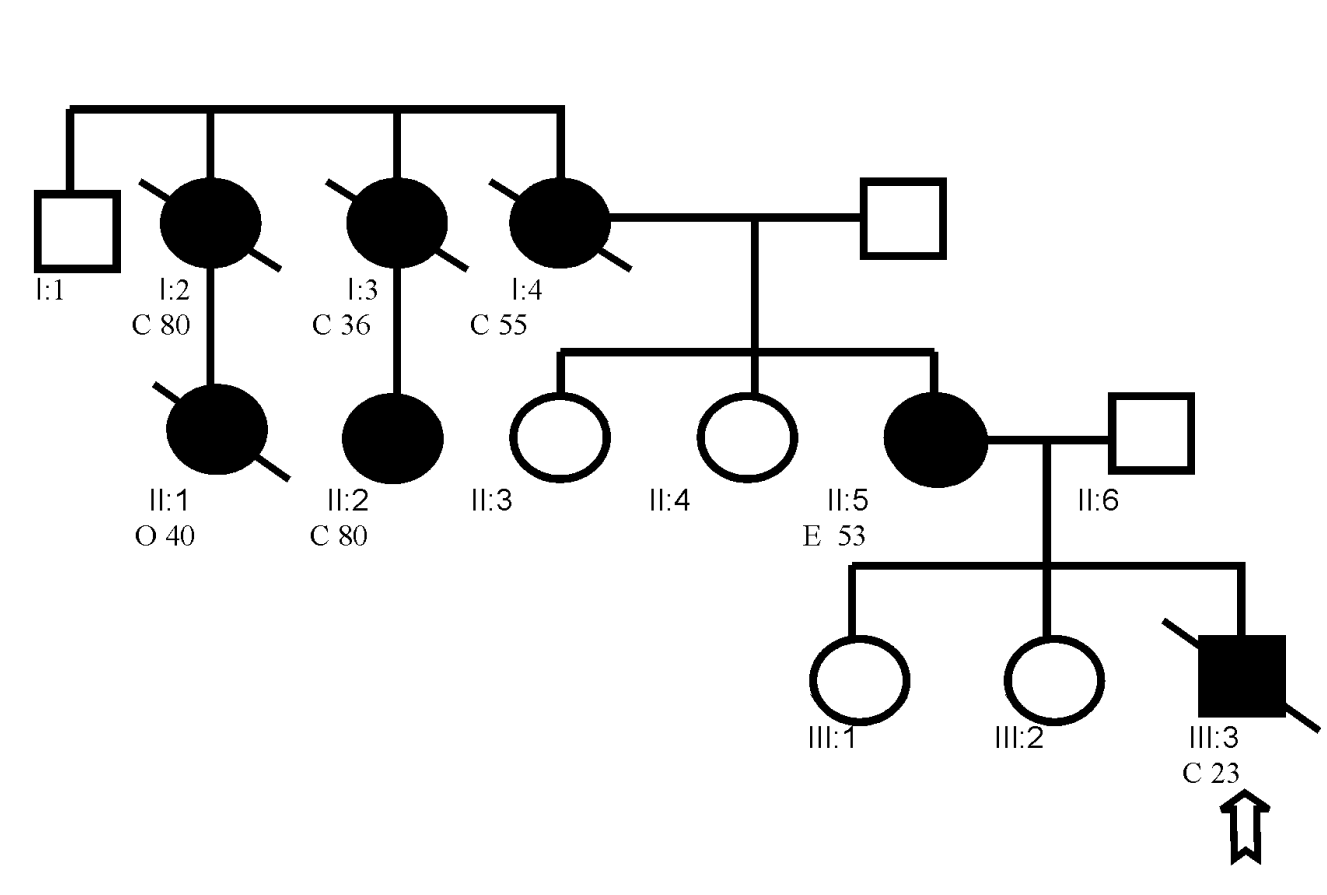
**Pedigree showing HNPCC family**. An arrow indicates the male index patient (III:3) diagnosed with colorectal adenocarcinoma at the age of 23 years. Family members suffering from a malignancy are indicated by a shaded circle or square. The age, type of malignancy, as well as the generation (roman figures), are described below the indicated patient. The family fulfill the Amsterdam-I criteria with presence of extracolonic tumors in the extended pedigree, having more than three carcinomas of colon (C) or ovary (O) in the affected members. The syndrome is present in all three generations (I-III) and three family members are younger than 50 years (III:3, II:1 and I:3). At the moment of the study the proband's mother (II-5) was an unaffected carrier, but two years later she developed an endometrial (E) adenocarcinoma.

The early onset of the colon cancer in the proband and the study of the family's pedigree (fig. 1), that fulfill the strict Amsterdam-1 criteria, prompted genetic analysis with suspicion of HNPCC. They were informed about the risks, benefits and limitations of the study protocol.

### Study protocol

As screening method for HNPCC we searched for MSI in the proband's primary tumor and metastasis tissue blocks. The absence of proband's non-tumor DNA for MSI testing was overcome studying the alleles carried by his progenitors. Once the MSI was established, a second blood sample was obtained from the proband's mother (obligate carrier but at the moment of the study still unaffected) to full mutation analysis of the *hMSH2 *and *hMLH1 *genes by direct sequencing. After identifying the family mutation, we searched it in both proband's sisters.

### DNA preparation for genetic testing

Peripheral blood was collected from the three consulting family members and the proband's father. Genomic DNA isolation from their lymphocytes was performed using a standard phenol-chloroform extraction. DNA from the proband was obtained from the formalin-fixed, paraffin embedded tissue blocks and isolated by microdissection of tumor, deparaffinization, proteinase K treatment, and ethanol precipitation [7].

### Determination of MSI

**Figure 2:**
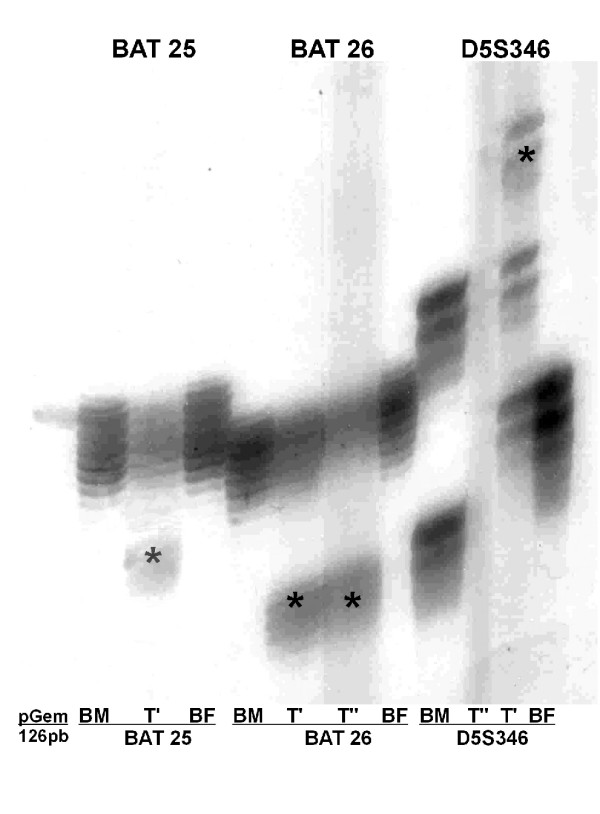
**Microsatellite instability**. Three of five microsatellite markers (BAT-25, BAT-26 and D5S346) presented different alleles (*) in the proband's primary tumor (T') and its metastasis (T") of those inherited from his biological mother (BM) and father (BF).

The reference panel of 5 microsatellite markers standardized by the National Cancer Institute for the screening of HNPCC (BAT-25, BAT-26, D2S123, D5S346 and D17S250) were PCR amplified using the corresponding specific primers for each one [8]. All the PCR products were electrophoresed through a denaturing 6% polyacrylamide gels and visualized by silver staining (fig. 2).

### DNA sequencing of hMSH2 and hMLH1 genes

A second blood sample was obtained from the proband's mother and forwarded to the Department of Clinical Cancer Genetics (City of Hope Cancer Center, Duarte, California, USA) to full mutation analysis of the *hMSH2 *and *hMLH1 *genes. The sample was amplified followed by direct sequencing to screen the coding regions of both the *hMSH2 *and the *hMLH1 *genes for germline mutation in the DNA.

After establishing the familial mutation in the proband's mother, located in the exon 12 of *hMSH2 *gene, it was searched in the proband's sisters by direct sequencing using a manual Sequenase PCR products kit (Amersham Biosciences).

## Results and discussion

Our work shows that the absence of proband's non-tumor DNA for MSI testing can be overcome by studying the alleles carried by his progenitors avoiding the need for initial sequencing of the obligate carrier.

Although BAT-26 has been reported to be sufficient for MSI-H detection even without normal tissue matching [9], careful interpretation is needed if MSI-H detection is based solely on this marker, since polymorphism at the BAT-26 locus has been detected [10].

A more sensitive approach has been reported using a quasimonomorphic mononucleotide markers panel (that includes BAT-25 and BAT-26) without the need to match normal DNA[11].

In the present case, we overcame the difficulty of having a proband post-mortem non-tumor tissue sample for MSI testing by studying the alleles carried by his progenitors. Microsatellites are inherited according to Mendelian rules like any other genetic markers. Each progenitor pass one of its two alleles to its offspring and by definition, the alleles present in the proband's tumor tissue but absent in his progenitors are the result of somatic mutation.

**Figure 3:**
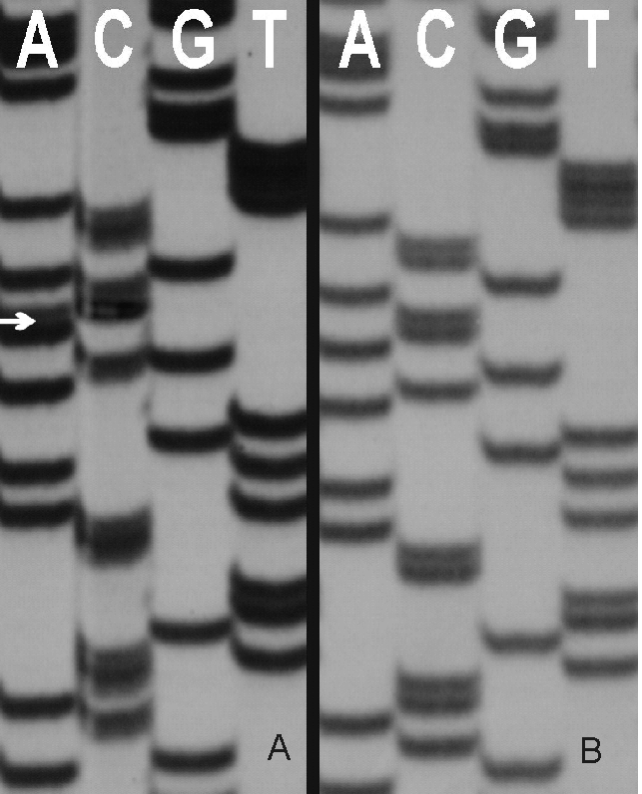
**DNA sequence analysis of *hMSH2 *exon 12**. Genomic DNA was isolated from leucocytes and PCR amplified with the help of *hMSH2 *exon 12 flanking primers. In the image the result of the sequencing using the PCR forward primer. Panel A: proband's mother (obligate carrier), positive for the mutation. Panel B: negative control for the mutation.

Three out of five microsatellite markers (BAT-25, BAT-26 and D5S346) presented alleles in the proband's primary tumor (T') and its metastasis (T") different from those inherited from his parents. This observation suggested a dysfunction of the mismatch repair system and the tumor was classified as high frequency MSI (MSI-H) according to the NCI workshop [12]. Direct sequencing of the *hMSH2 *and *hMLH1 *genes was indicated, detecting a novel germline mutation, a c.1864C>A transversion in exon 12 of *hMSH2 *gene at the heterozygous state (fig. 3) leading to a proline 622 to threonine (p.Pro622Thr) amino acid substitution. This is the second report involving the 622 codon in HNPCC [13].

**Figure 4:**
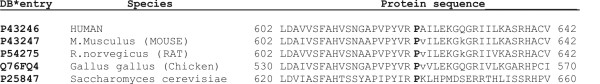
**Protein sequence alignment for hMSH2 and homologues**. Human, Mouse, Rat, Chicken and Saccharomyces cerevisiae (the site of mutation is highlighted) protein sequence alignment. Evolutionary conservation may indicate the functional relevance of the aminoacid involved for the structure or functioning of the protein. *UniProtKB/Swiss-Prot

Evolutionary conservation, examined by alignment of sequences of homologous proteins for several species (fig. 4), suggests a functional relevance for the amino acid involved. This is also supported by the mutator phenotype described for Pro640Leu mutant yeast [14], homologous to Pro622Leu *hMSH2 *substitution in humans (fig. 4).

Mutation screening was done in both sisters to assign their risk to develop HNPCC and other related cancers. We found the mutation in one of them and opportune recommendations for surveillance and prophylaxis were given. Two years after that, the proband's mother, unaffected during this study, developed an endometrial adenocarcinoma.

## Conclusion

We overcame the absence of proband's non-tumor DNA for MSI testing by studying the alleles carried by his progenitors, the natural approach for the research team due its paternity testing background.

This strategy allowed us to perform the screening for HNPCC using the recommended NCI microsatellite panel before sequencing the obligate carrier.

We consider highly probable the disease-causing nature of the germline mutation in the *hMSH2 *gene found in the family. To establish it undoubtedly, both immunohistochemical data of the investigated tumor and screening of at least 100 chromosomes in healthy controls should be performed. This is the second report of an HNPCC related mutation in Argentina, involving the hMSH2 gene [15]. According to the Human Gene Mutation Database (HGMD) [16] and the International Society for Gastrointestinal Hereditary Tumors (InSiGHT) [17] Database we are the first to report the mutation.

## Competing interests

The author(s) declare that they have no competing interests.

## Authors' contributions

EAS and SGC designed and directed the study. C.F., C.L. R.D. and A.S. performed the molecular analyses. DD wrote the manuscript.

## Pre-publication history

The pre-publication history for this paper can be accessed here:


